# Supplementary material for: Akt inhibition improves long‐term tumour control following radiotherapy by altering the microenvironment
Source: EMBO Mol Med. 2017 Oct 30;9(12):1646–59. doi: 10.15252/emmm.201707767 (PMC5709765; doi:10.15252/emmm.201707767)
Supplement: Supplementary file 1 — Appendix [file EMMM-9-1646-s001.pdf]

Appendix

1. **Appendix figures S1-S5**
2. **Appendix tables S1-S4**

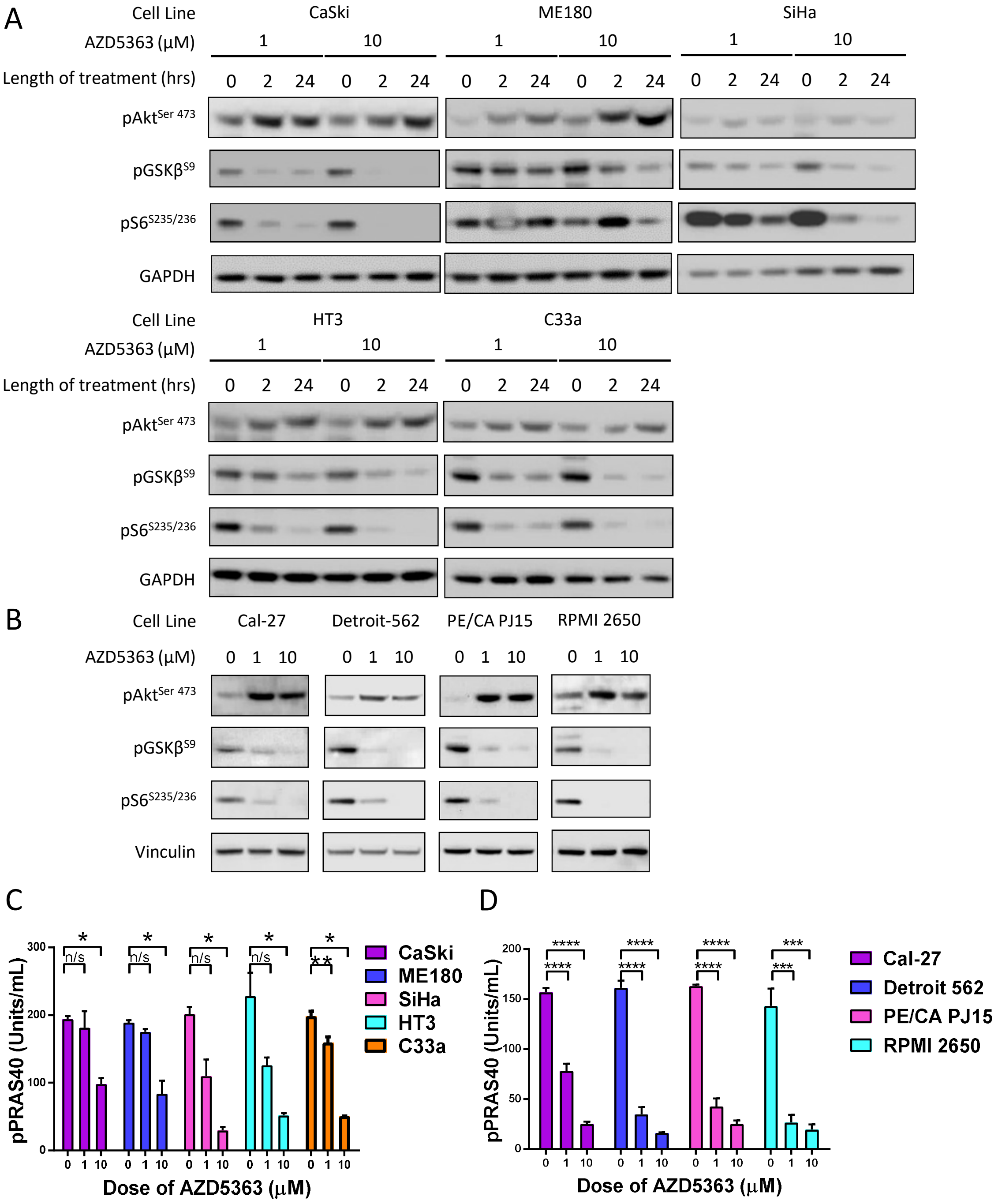

A
Cell Line
CaSki
ME180
SiHa
AZD5363 (μM)
1
10
Length of treatment (hrs)
0 2 24 0 2 24
pAkt Ser 473
pGSKβ S9
pS6 S235/236
GAPDH
HT3
C33a
B
Cal-27
Detroit-562
PE/CA PJ15
RPMI 2650
0 1 10
Vinculin
C
pPRAS40 (Units/mL)
Dose of AZD5363 (μM)
n/s
CaSki
ME180
SiHa
HT3
C33a
D
Cal-27
Detroit 562
PE/CA PJ15
RPMI 2650

Figure S1.

Akt inhibition with AZD5363 can be demonstrated in a wide range of cancer cells *in vitro.*

A-D. Cells of differing origins were treated with 1 µM or 10 µM AZD5363 for 2 hours or 24 hours (A), or 2 hours only (B-D), before lysis on ice; (***n*** = 3 experiments). (A-B), Western blots were performed with antibodies detecting pAkt, pGSK3β, pS6 and the housekeeping protein GAPDH or vinculin. A representative is shown for each cell line, where (A) shows cells of a head and neck cancer origin, and (B), cells of a cervical cancer origin. (C-D), ELISA was used to measure levels of pPRAS40.

Data information: in (C) and (D) data is shown as mean ± SEM; n/s ***P*** > 0.05, * ***P*** < 0.05, ** ***P*** < 0.01, *** ***P*** < 0.001, **** ***P*** < 0.0001). Statistical test is one-way ANOVA with Dunnett's *post hoc* test (for (C); ***P*** = 0.001-0.834, and for (D); ***P*** = 0.0001-0.0007).

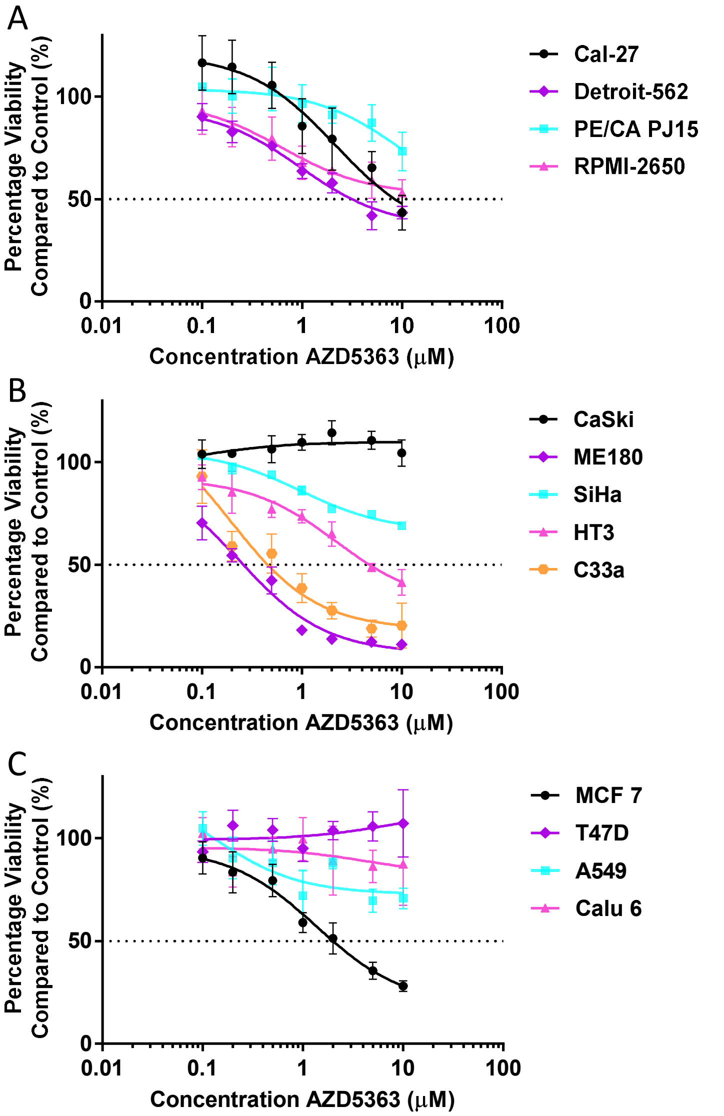

Figure S2.

AZD5363 has a cell line dependent effect on viability *in vitro.*

A-C. Cell lines of head and neck cancer (A), cervical cancer (B) and breast and lung cancer (C) origin, were treated with 1-10μM AZD5363 for 96hrs before an MTT assay was performed; (***n*** = 3 experiments).

Data information: Results are displayed as mean ± SEM, normalised to a DMSO treated control and fitted to a dose-response curve.

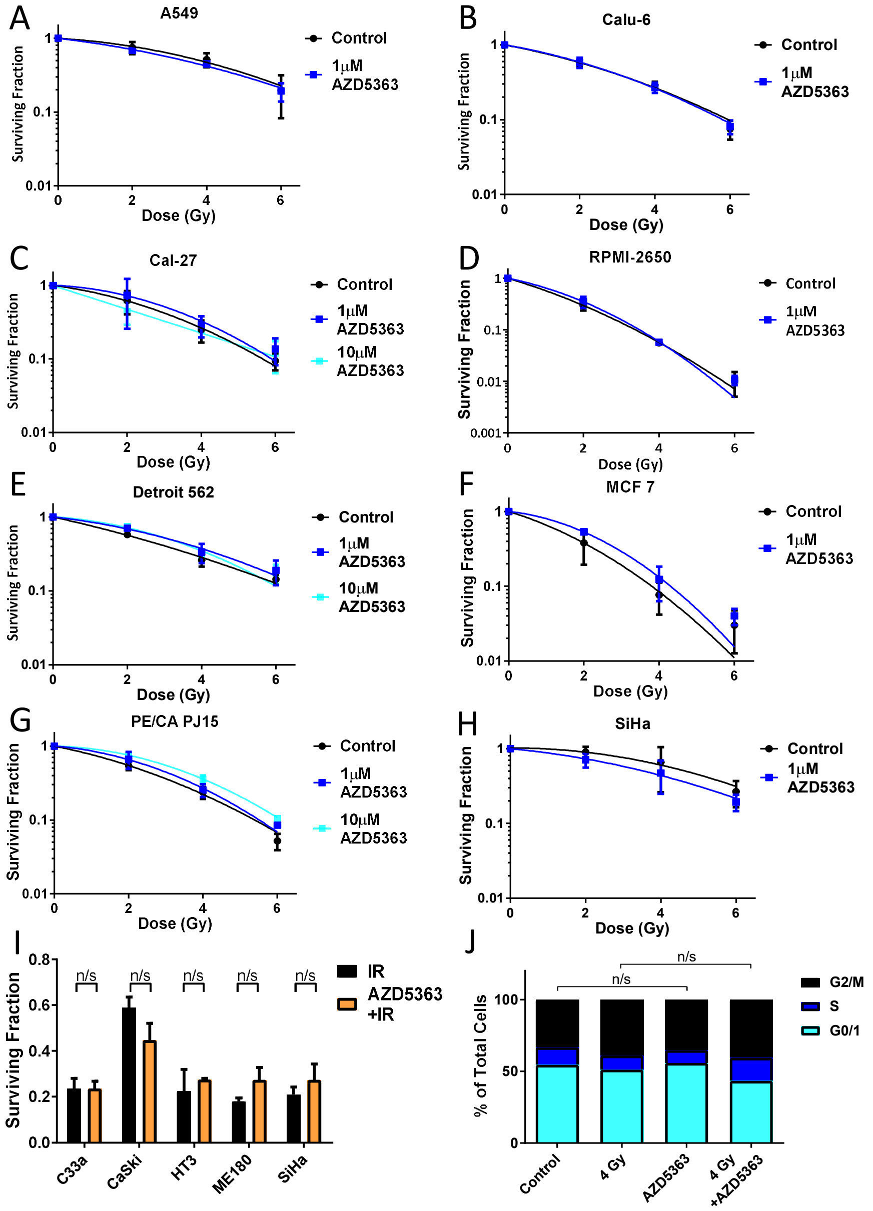

A
A549
Surviving Fraction
Dose (Gy)
Control
1μM AZD5363
B
Calu-6
Control
1μM AZD5363
C
Cal-27
Control
1μM AZD5363
10μM AZD5363
D
RPMI-2650
Control
1μM AZD5363
E
Detroit 562
Control
1μM AZD5363
10μM AZD5363
F
MCF 7
Control
1μM AZD5363
G
PE/CA PJ15
Control
1μM AZD5363
10μM AZD5363
H
SiHa
Control
1μM AZD5363
I
n/s
IR
AZD5363 +IR
C33a
CaSki
HT3
ME180
SiHa
J
% of Total Cells
G2/M
S
G0/1
Control
4 Gy
AZD5363
4 Gy +AZD5363

Figure S3.
Akt inhibition with AZD5363 does not enhance the radiosensitivity of tumour cells *in vitro* and has no effect on the cell cycle of FaDu cells with or without radiation*.*
A-H. Clonogenic assay of (A) A549, (B) Calu-6, (C) Cal-27, (D) RPMI-2650, (E) Detroit 562, (F) MCF 7, (G) PE/CA PJ15 and (H) SiHa cells treated with 1 μM or 10 μM AZD5363 for 2 hours before, and 24 hours after a single dose of RT (2, 4 or 6 Gy); (***n*** = 3 experiments).
I. Surviving fraction of cells of a cervical cancer origin after a single 4 Gy dose of RT combined with 10 μM AZD5363; (***n*** = 3 experiments).
J. Flow cytometric analysis of FaDu cell cycle with propidium iodide which shows no effect of 24hrs 1 μM AZD5363 either alone or after 4 Gy irradiation; (***n*** = 3 experiments).
Data information: In (A-H) values are shown of surviving fraction (mean ± SEM) and are fitted to the linear quadratic model. In (I) values are of surviving fraction (mean ± SEM). Statistical test is unpaired t-test, one per cell line, (***P*** = 0.163-0.844). In (J) stacked bars represent mean percentage total cells in each treatment group; n/s ***P*** > 0.05. Statistical test is Kruskal-Wallis with Dunn's *post hoc* test (***P*** = > 0.9999).

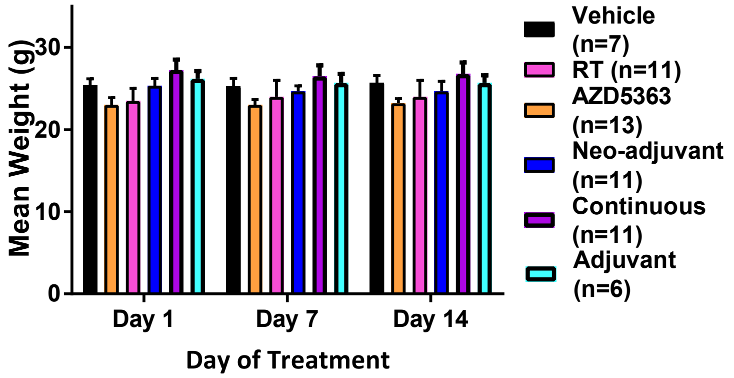

Figure S4.
The combination of RT with AZD5363 does not cause loss of animal weight.
A. Mean weights of FaDu tumour bearing mice treated with AZD5363 (50 mg/kg BD) or 6 Gy RT alone, or in combination, with the drug given as an adjuvant, continuously, or as a neo-adjuvant.
Data information: Plots show mean weights and are the combined data of 2 independent experiments; (***n*** = 6-13 mice/experiment).

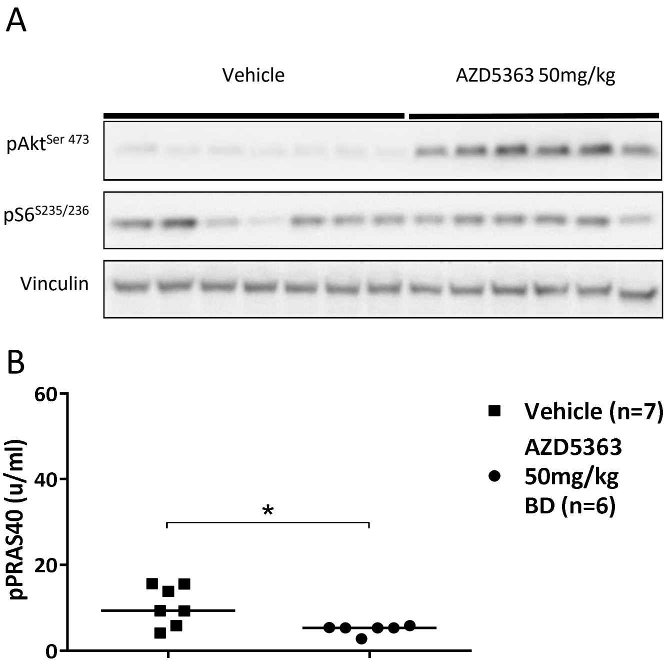

Figure S5.
AZD5363 targets the Akt pathway when used *in vivo*.
A-B. FaDu tumour bearing mice were treated with AZD5363 (50 mg/kg) for 7 days (14 doses) after which mice were culled and lysates prepared; (***n*** = 6-7 per group). (A), Western blot with antibodies detecting pAkt, pS6 and the housekeeping protein vinculin. (B), pPRAS40 levels as measured by ELISA; * ***P*** < 0.05.
Data information: In (B) data shows individual results with line to represent median value. Statistical test is Mann-Whitney test (* ***P*** = 0.032).

Table S1- Cell lines and culture requirements

Cell Line	Origin	Source	Media
**FaDu**	H&N	ATCC	Advanced MEM<br>+10%FCS<br>+ 10ml 100x L-Glutamine
**CAL-27**	H&N	ATCC	DMEM<br>+10%FCS<br>+ 10ml 100x L-Glutamine
**Detroit-563**	H&N	ATCC	EMEM<br>+10%FCS<br>+ 10ml 100x L-Glutamine
**RPMI 2650**	H&N	ATCC	EMEM<br>+10%FCS<br>+ 10ml 100x L-Glutamine
**PE/CA PJ15**	H&N	ECACC	IMDM<br>+10%FCS<br>+ 10ml 100x L-Glutamine
**PE/CA PJ34 (Clone C12)**	H&N	ECACC	IMDM<br>+10%FCS<br>+ 10ml 100x L-Glutamine
**ME-180**	Cervical	ATCC	DMEM<br>+10%FCS<br>+ 10ml 100x L-Glutamine
**Ca Ski**	Cervical	ATCC	RPMI-1640<br>+10%FCS<br>+ 10ml 100x L-Glutamine
**SiHa**	Cervical	ATCC	EMEM<br>+10%FCS<br>+ 10ml 100x L-Glutamine
**C-33 A**	Cervical	ATCC	EMEM<br>+10%FCS<br>+ 10ml 100x L-Glutamine
**HT3**	Cervical	ATCC	DMEM<br>+10%FCS<br>+ 10ml 100x L-Glutamine
**MCF7**	Breast	ATCC	EMEM<br>+10%FCS<br>+ 10ml 100x L-Glutamine
**T-47D**	Breast	ATCC	RPMI-1640<br>+10%FCS<br>+ 10ml 100x L-Glutamine
**A549**	Lung	ATCC	F12<br>+10%FCS<br>+ 10ml 100x L-Glutamine
**Calu-6**	Lung	ATCC	EMEM<br>+10%FCS<br>+ 10ml 100x L-Glutamine
**HUVEC**	Human Umbilical Vein Endothelial Cells	Millipore	EndoGRO-LS Complete Culture Media Kit

Abreviations: H&N, Head and Neck; FCS, Foetal Calf Serum; MEM, Minimum Essential Medium; DMEM, Dulbecco's Modified Eagle Medium; EMEM, Eagle's Minimum Essential Medium; IMDM, Iscove's Modified Dulbecco's Medium; RPMI-1640, Roswell Park Memorial Institute 1640 Medium; Suppliers: ATCC, American Type Culture Collection (Manassas, USA); ECACC, European Collection of Authenticated Cell Cultures (Salisbury, UK); Millipore (Watford, UK).

Table S2- Antibodies used in Western blot analysis

Antibody	Supplier	Species	Size (kDa)	Dilution
**Phospho-Akt (Ser473)**	Cell Signaling Technology, Danver, USA	Rabbit	66	1:500
**Phospho-GSK3β (Ser9)**	Cell Signaling Technology, Danver, USA	Rabbit	46	1:500
**Phospho-S6 Ribosomal Protein (Ser235/236)**	Cell Signaling Technology, Danver, USA	Rabbit	32	1:1000
**Vinculin**	Sigma-Aldrich, Dorset, UK	Mouse	116	1:5000
**GAPDH**	Sigma-Aldrich, Dorset, UK	Rabbit	37	1:10000
**Anti-rabbit IgG, HRP-linked Antibody**	Cell Signaling Technology, Danver, USA	Goat	N/A	1:2000
**Anti-mouse IgG, HRP-linked Antibody**	Cell Signaling Technology, Danver, USA	Horse	N/A	1:2000

Table S3- ELISA kits used

ELISA Kit	Supplier
**PRAS40 (pT246) Phospho-ELISA Kit (Human)**	Invitrogen (Paisley, UK).
**Human VEGF Quantikine® ELISA Kit**	R&D (Abingdon, UK)
**Mouse VEGF Quantikine® ELISA Kit**	R&D (Abingdon, UK)

Table S4- Summary of Immunohistochemical staining techniques

Stain	Antigen Retrieval	Blocking Steps	Primary Antibody (supplier*, dilution and incubation)	Detection (supplier*)
**CD31**	HIER (100°C, pH6, 20mins)	EPB, 10mins<br>PPB, 30mins	Anti-CD31 antibody, rabbit polyclonal (Abcam, 1:225, 15mins, RT)	NovoLink Polymer Refine Kit (Leica)
**HIF-1α**	HIER (100°C, pH9, 40mins)	EPB, 10mins<br>PPB, 30mins<br>BS, 30 mins	Anti-HIF-1-alpha antibody [EP1215Y], rabbit mAb (Abcam, 1:1000, 60mins, RT)	NovoLink Polymer Refine Kit (Leica)
**Human Ki-67**	HIER (125°C 1min & 90°C 10sec, pH6)	EPB, 10mins<br>BR, 20mins	Monoclonal Mouse Anti-Human Ki-67 Antigen Clone MIB-1 (Dako, 1:100, 20mins, RT)	ARK™ (Animal Research Kit) Peroxidase, (Dako)
**Pimonidazole**	HIER (125°C 1min & 90°C 10sec, pH6)	EPB, 10mins<br>CB, 20mins	Affinity purified rabbit anti- pimonidazole antibody (Hydroxyprobe, 1:300, 120mins, RT)	EnVision-HRP Kit (Dako)
**CD11b**	HIER (pH6, 95°C 32mins)	DI, 8mins	Anti-CD11b antibody [EPR1344], rabbit mAb (Abcam, 1:4000, 40mins, 37°C)	DISCOVERY® OmniMap anti-Rabbit HRP (Ventana)
**Mouse Ki-67**	HIER (pH8.5, 95°C, 64 mins	DI, 8mins	Ki-67, rabbit anti-mouse (Bethyl, 1:2000, 60mins, 37°C)	DISCOVERY® OmniMap anti-Rabbit HRP (Ventana)

Abreviations: HIER, Heat-induced Epitope Retrieval; EPB, Endogenous Peroxidase Block (3% H_2O_2, Sigma); PPB, Post Primary Block (Leica Refine Kit); BS, Blocking Solution (Candor); BR, Blocking Reagent (normal mouse serum, Dako); CB, 10% Casein Block (Vector); DI, Discovery Inhibitor (Ventana); RT, Room Temperature; mAb, monoclonal Antibody. *Suppliers: Abcam (Cambridge, UK); Dako (Ely, UK); Vector Laboritories (Peterborough, UK); Leica, Leica Microsystems (Milton Keynes, UK); Ventana (Tucson, USA); Hydroxyprobe (Burlington, USA); Candor, (Candor Bioscience, Wangen, Germany); Sigma, Sigma-Aldrich (Dorset, UK); Dako (Ely, UK); Bethyl (Bethyl Laboratories, Montgomery, USA).
