# Supplementary material for: Akt inhibition improves long‐term tumour control following radiotherapy by altering the microenvironment
Source: EMBO Mol Med. 2017 Oct 30;9(12):1646–59. doi: 10.15252/emmm.201707767 (PMC5709765; doi:10.15252/emmm.201707767)
Supplement: Supplementary file 2 — Expanded View Figures PDF [file EMMM-9-1646-s002.pdf]

Expanded View Figures

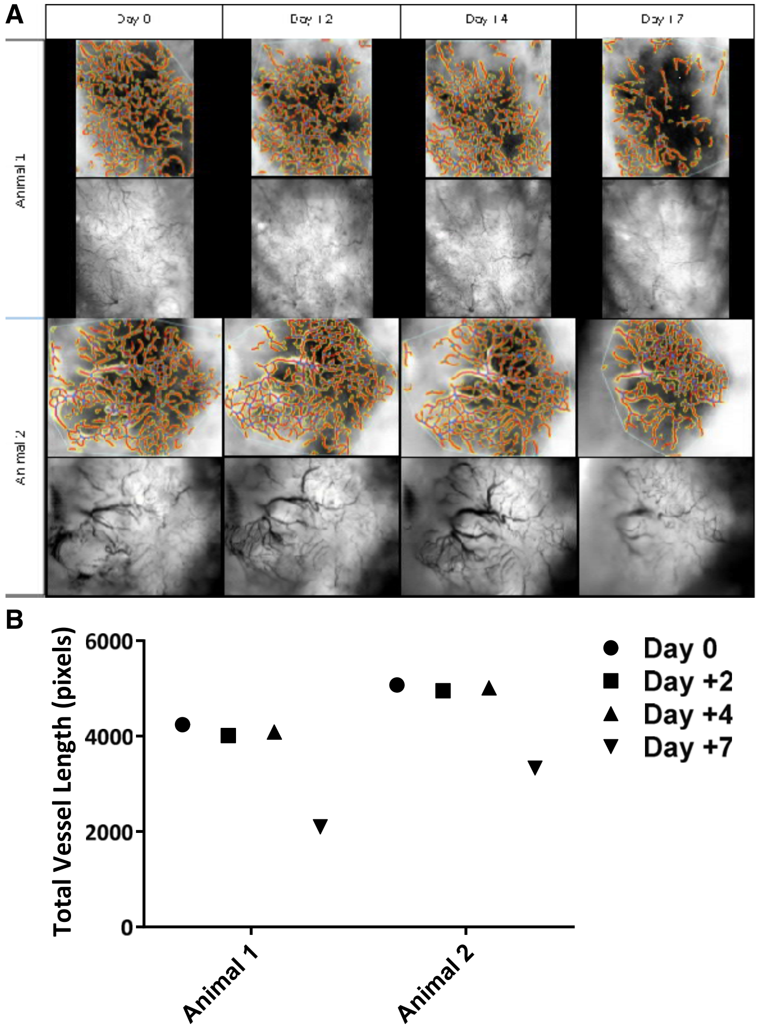

Figure EV1. A reduction in total vessel length occurs 7 days post-RT in mice receiving adjuvant AZD5363.

Two mice bearing FaDu tumours grown within dorsal windows were treated with 6 Gy RT and adjuvant AZD5363 (50 mg/kg BD), commencing on the first-day substantial tumour vasculature was visualised post-tumour inoculation (termed day 0).

A Mice were imaged using bright-field microscopy on days 2, 4 and 7 following RT.

B Total vessel length estimated with the assistance of computer software.

© 2017 The Authors

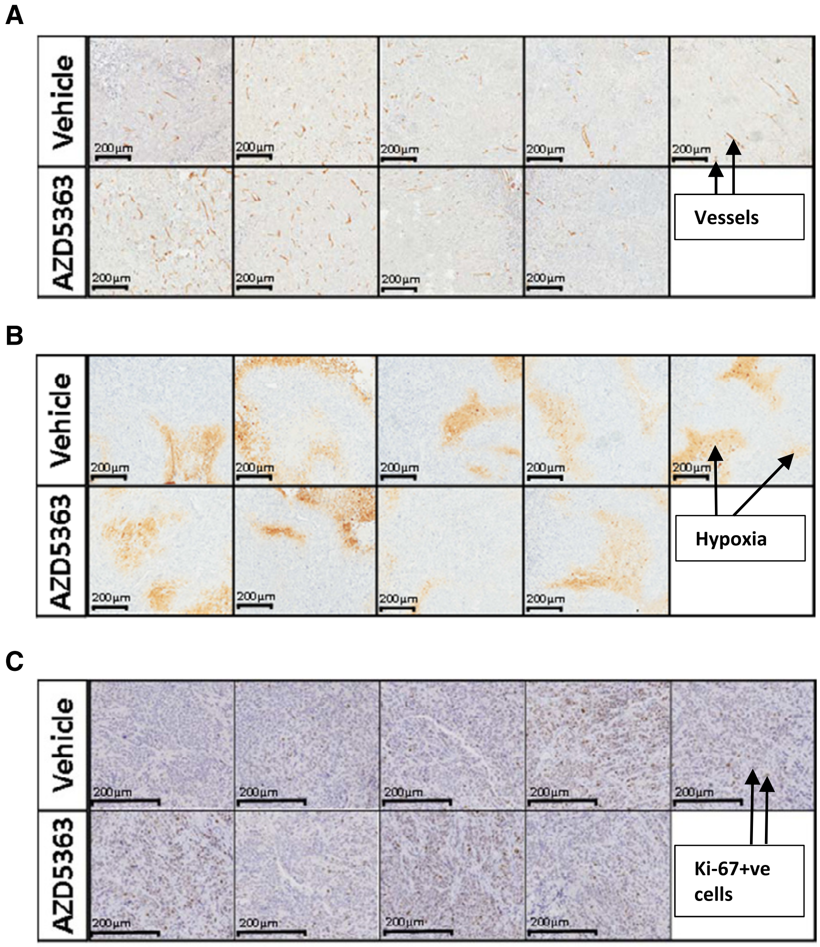

Figure EV2. AZD5363 does not reduce vascularity, increase hypoxia or alter tumour cell proliferation when given as a single agent.

FaDu tumour-bearing mice were treated with AZD5363 (50 mg/kg) for 7 days (14 doses) after which mice were culled and the tumours excised and prepared for histological analysis. Tumour sections were stained with antibodies to detect CD31, pimonidazole and Ki-67. Three sections were stained with each antibody, per tumour; *n* = 4–5/group.

A CD31 staining to allow visualisation of tumour vessels, 10× magnification.
B Pimonidazole staining to allow estimation of tumour hypoxia, 10× magnification.
C Ki-67 staining to assess tumour cell proliferation, 10× magnification.

 © 2017 The Authors

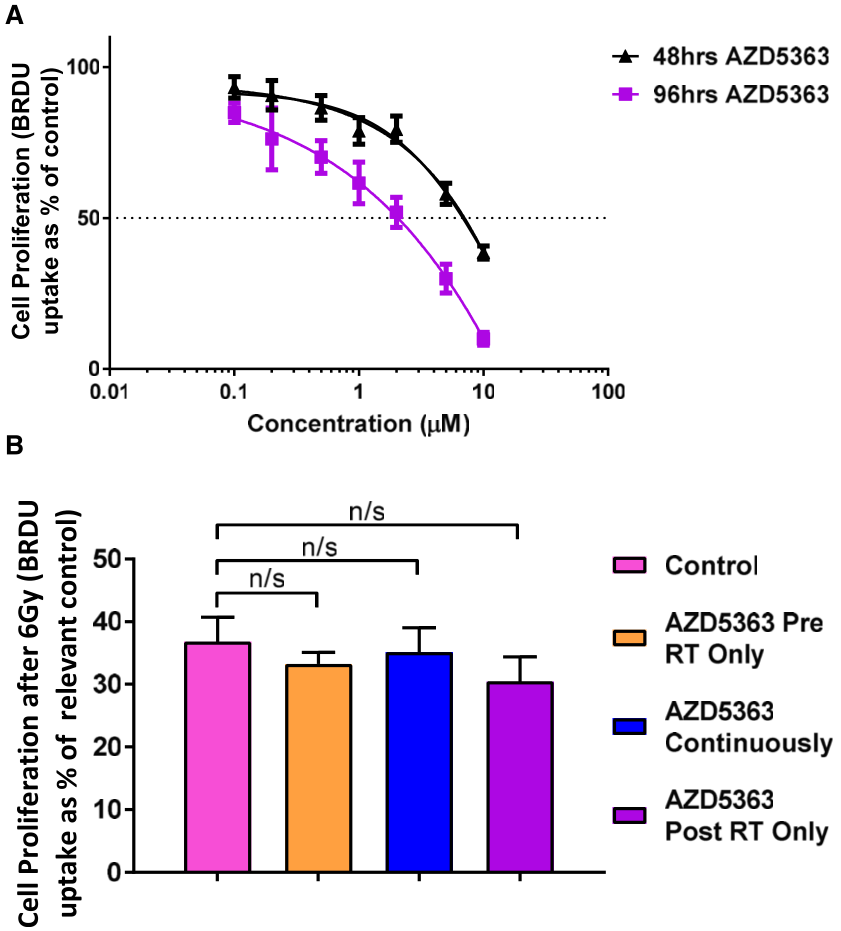

Figure EV3. AZD5363 reduces human vascular endothelial cell proliferation but does not cause a greater than additive effect on proliferation after RT.

- A HUVEC cells (1,000 cells/well) were seeded in a gelatin-coated 96-well plate and treated with doses of AZD5363 ranging from 0.1 to 10 µM for either 48 or 96 h. BrdU was added overnight, and an ELISA then performed to detect BrdU incorporation. A greater than 50% reduction in proliferation is produced with AZD5363 at a dose of between 2 and 3 µM (n = 3 experiments).
- B A BrdU assay was performed as in (A) but with the addition of 1 µM AZD5363 for 2 h before, 2 h before and 96 h after, or for 96 h after, a single 6-Gy dose of RT. None of the treatment schedules produced a greater than additive effect on the proliferation of vascular endothelial cells 96 h after a single 6-Gy dose of RT.

Data information: In (A) results are shown as the mean ± SEM, normalised to DMSO-treated control data and fitted to a dose-response curve. In (B) data is shown as the mean ± SEM, normalised to the relevant AZD5363 alone treated control; n/s = $P >$ 0.05. Statistical test is Kruskal-Wallis with Dunn's *post hoc* test (adjusted P = 0.523 to >0.9999).

© 2017 The Authors
