# Supplementary material for: Akt inhibition improves long‐term tumour control following radiotherapy by altering the microenvironment
Source: EMBO Mol Med. 2017 Oct 30;9(12):1646–59. doi: 10.15252/emmm.201707767 (PMC5709765; doi:10.15252/emmm.201707767)

C33a and Siha (n=3) pAkt, pGSK3B, pS6

15 seconds

450 seconds

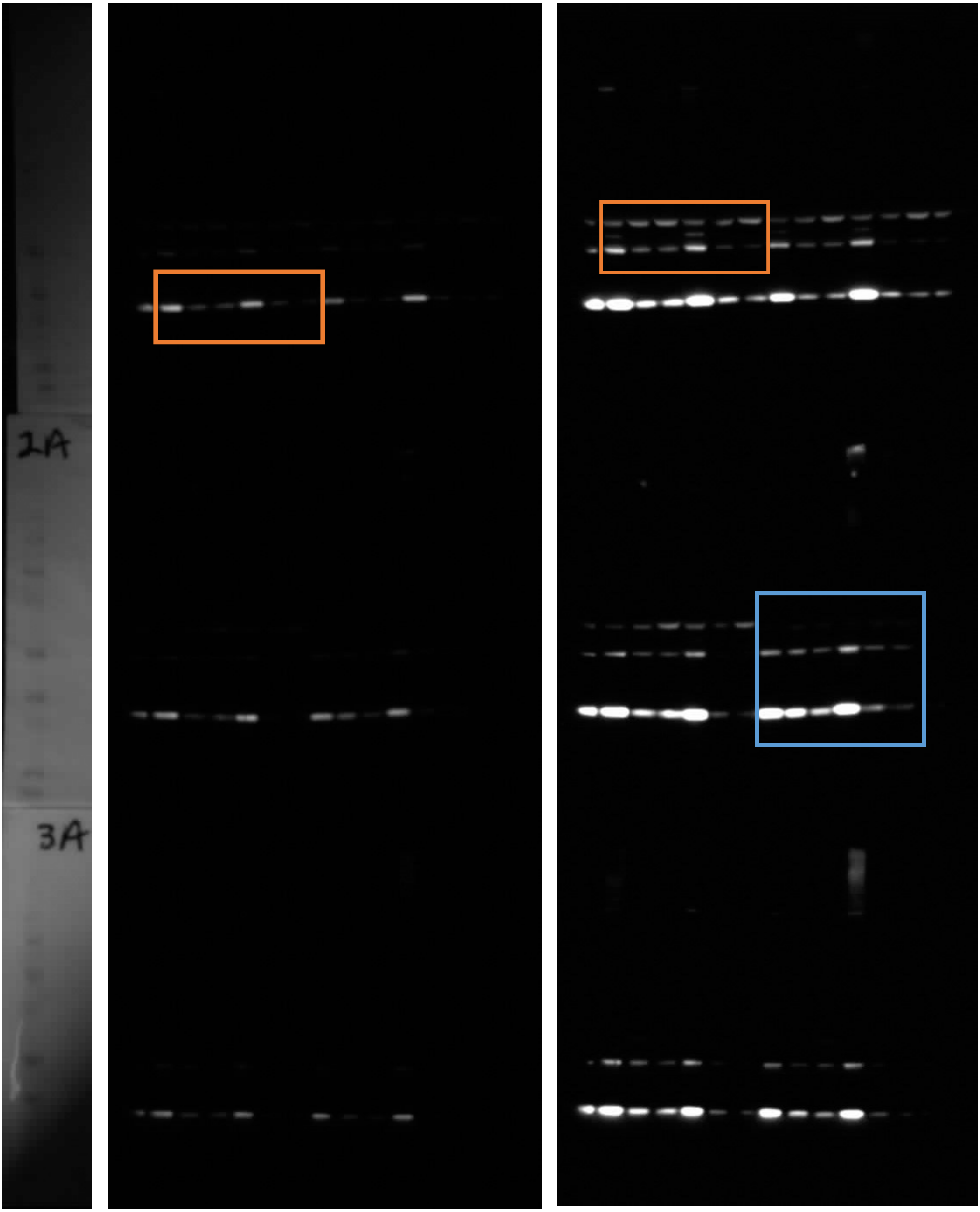

C33a and Siha (n=3) GAPDH

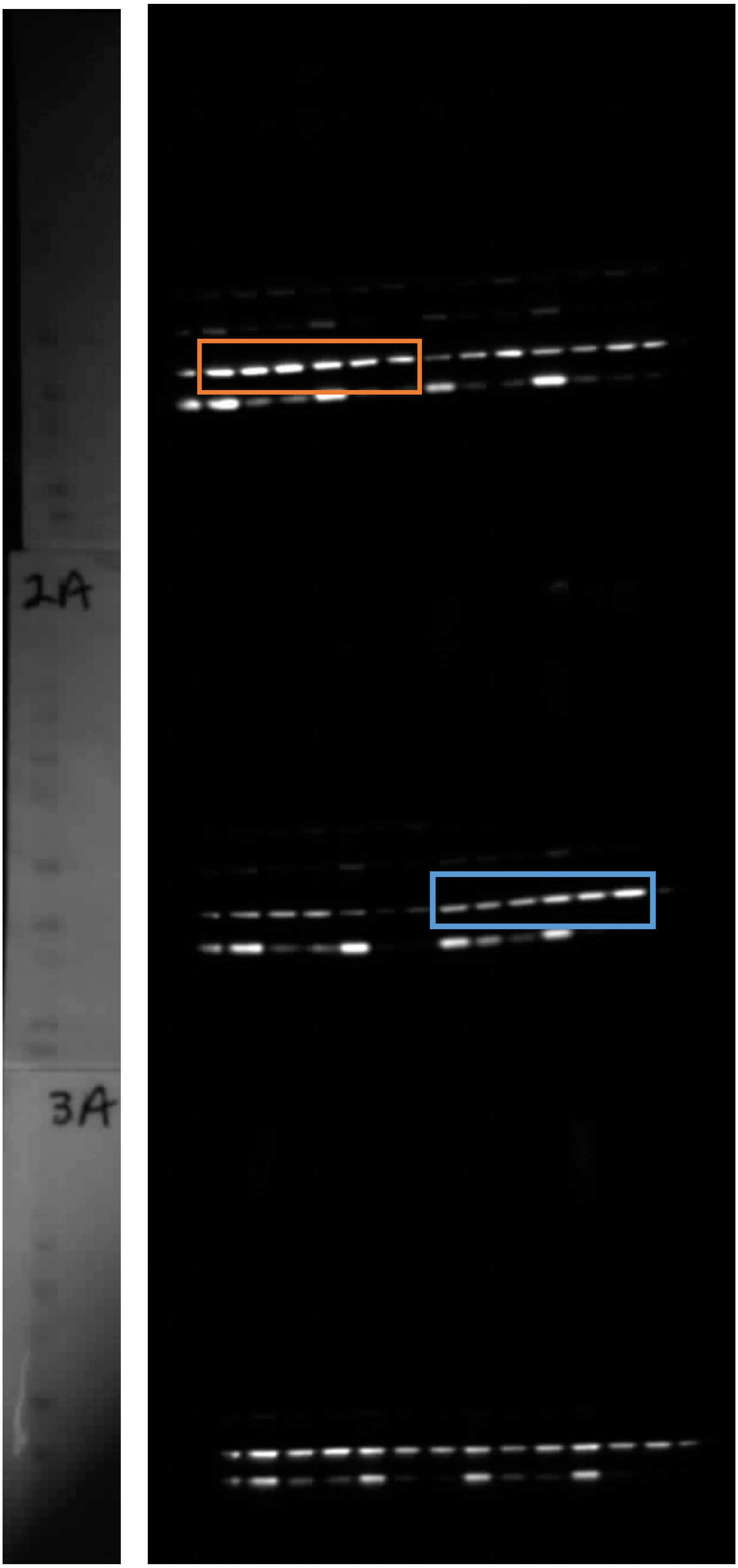

CaSki and HT3 (n=3) pAkt, pGSK3B, pS6

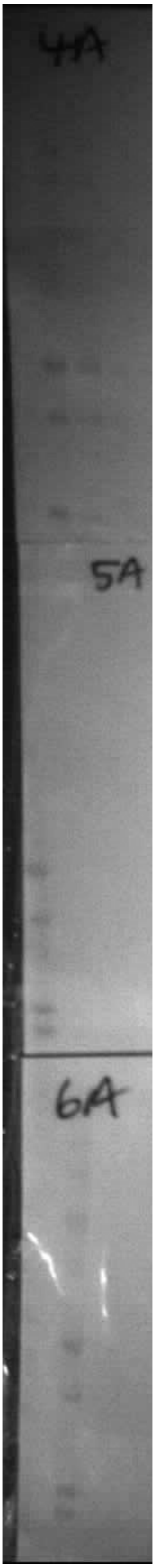

14 minutes

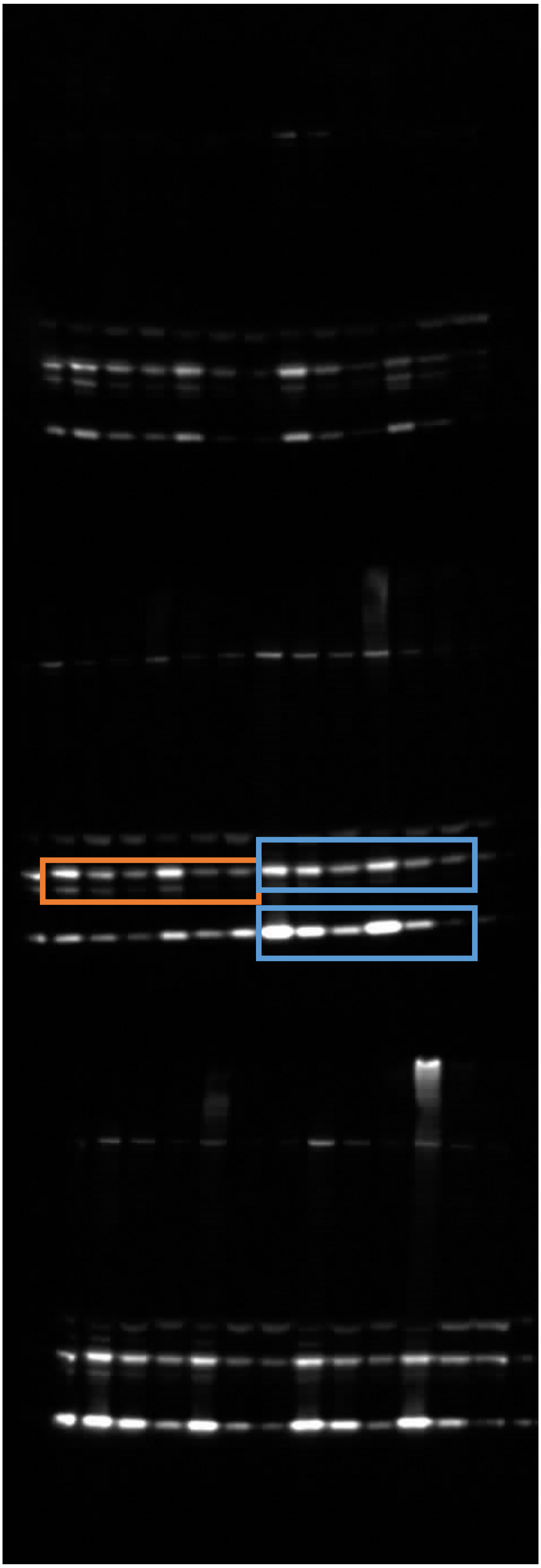

26 minutes

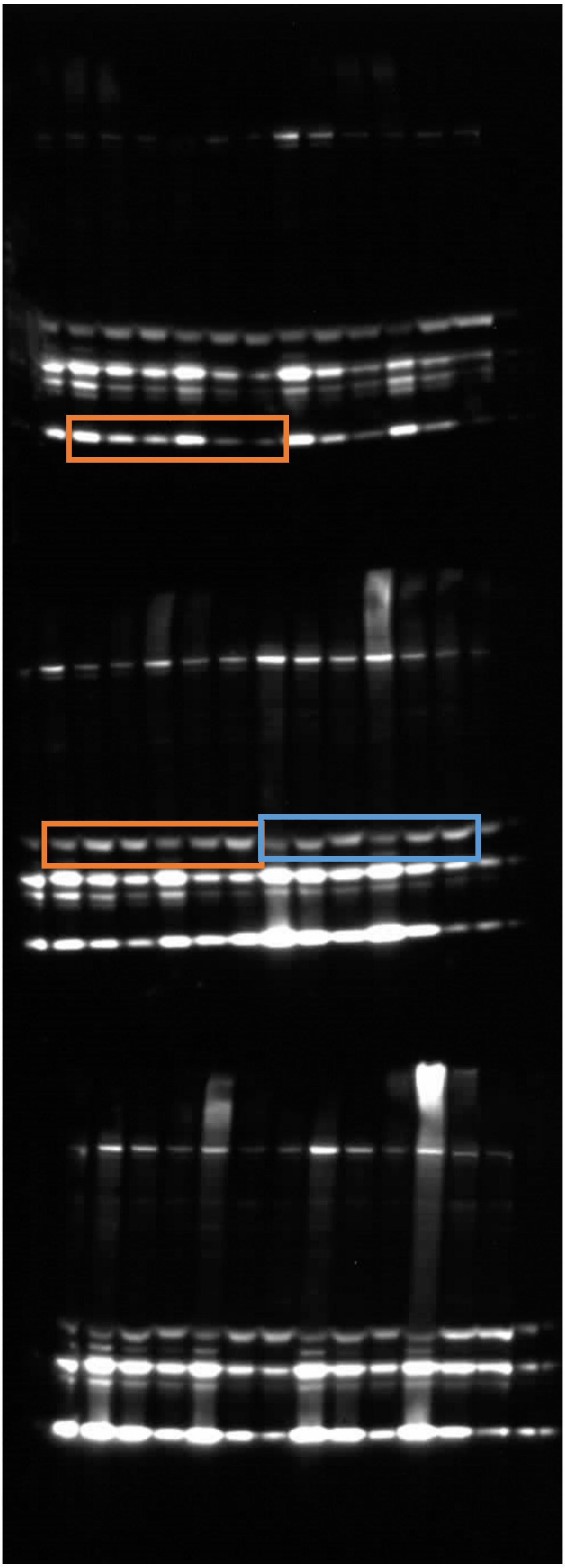

CaSki and HT3 (n=3) GAPDH

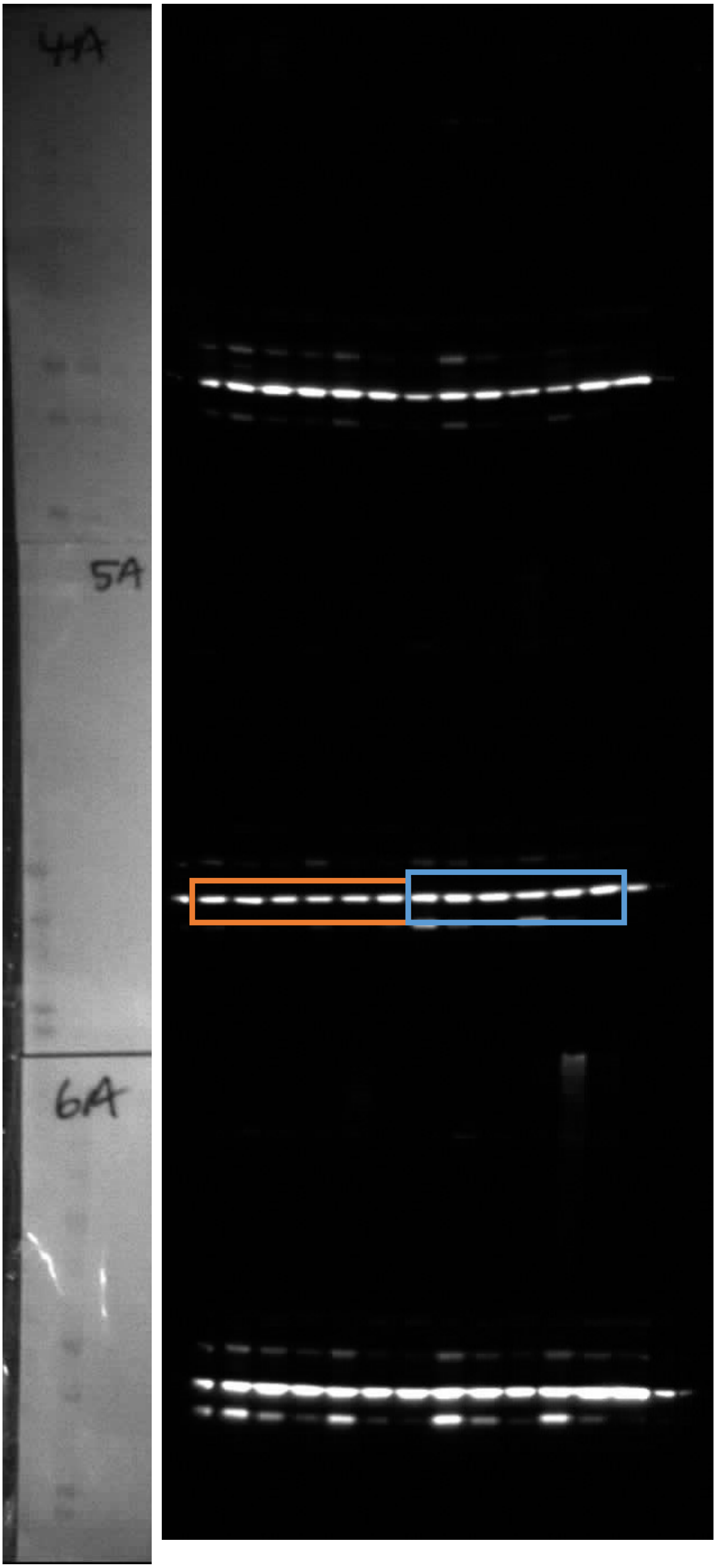

ME180 (n=3) pAkt, pGSK3B, pS6

12 minutes 26 minutes

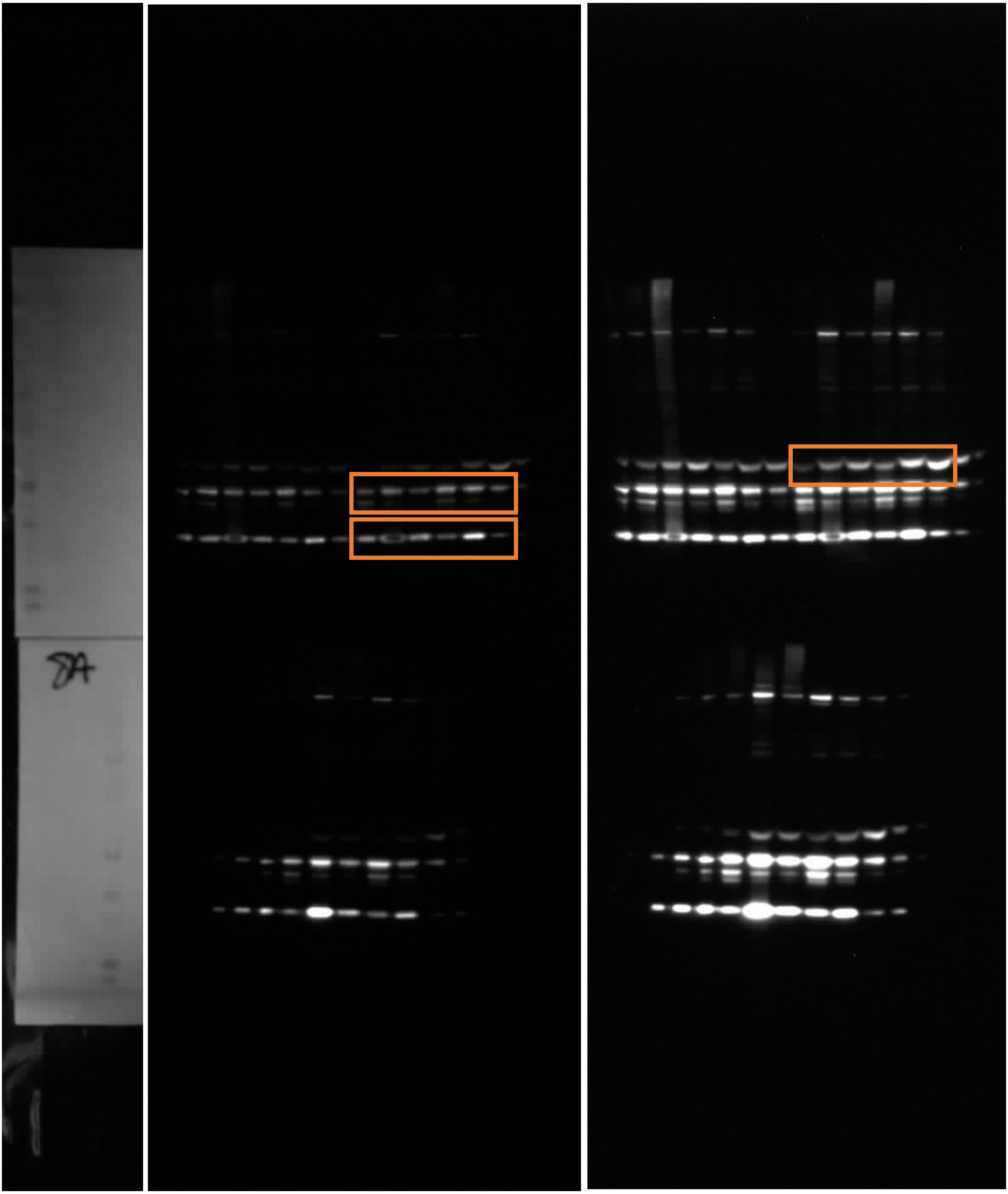

ME180 (n=3) GAPDH

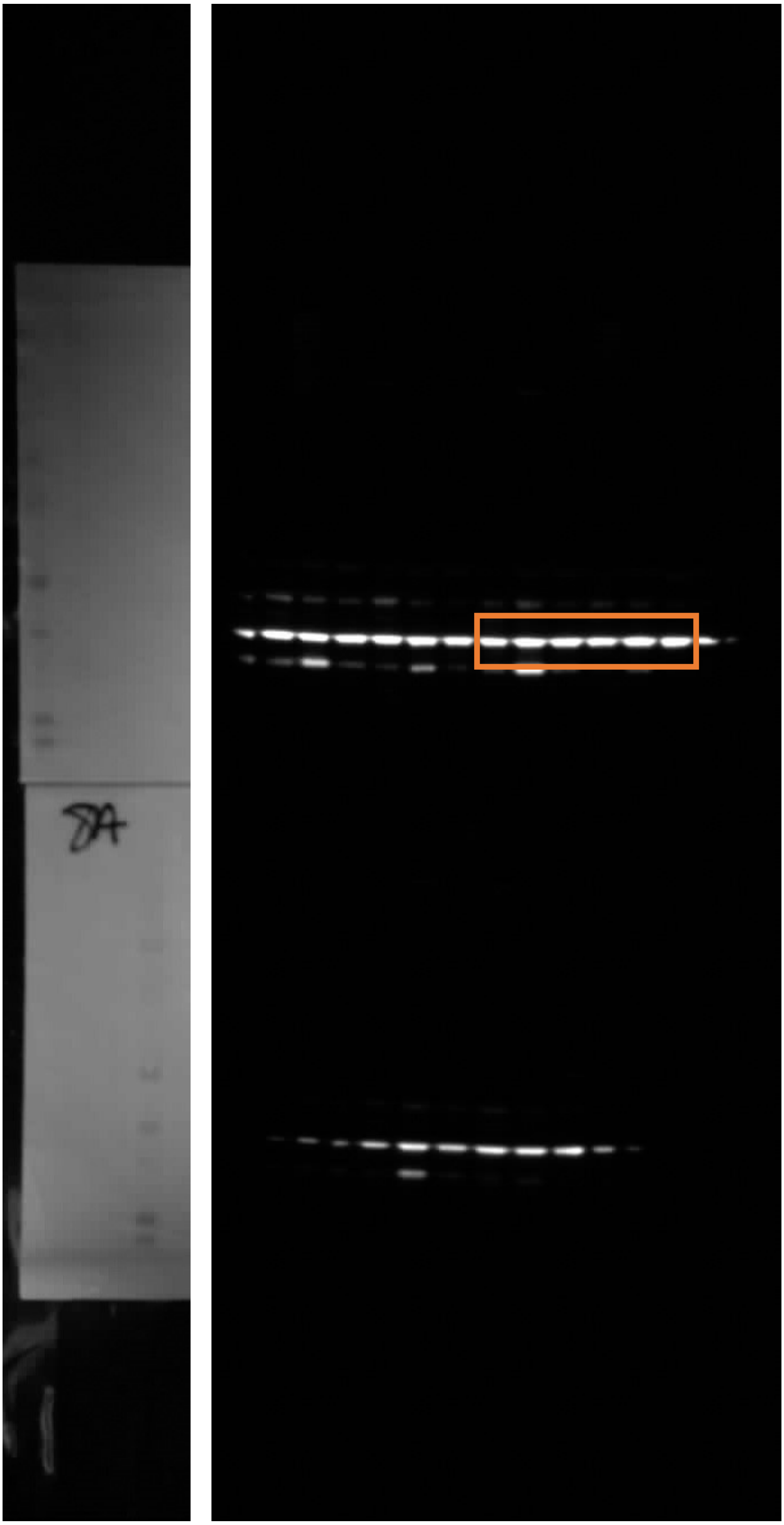

Cal 27 (n=3)

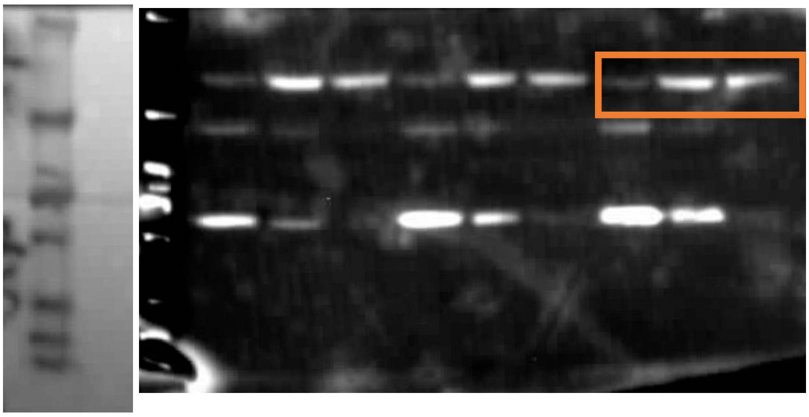

pAkt

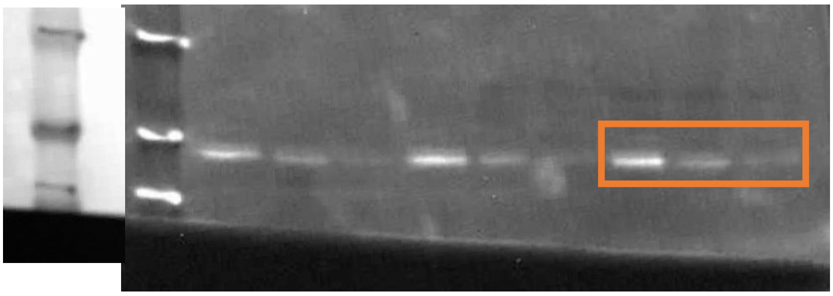

pGSK3B

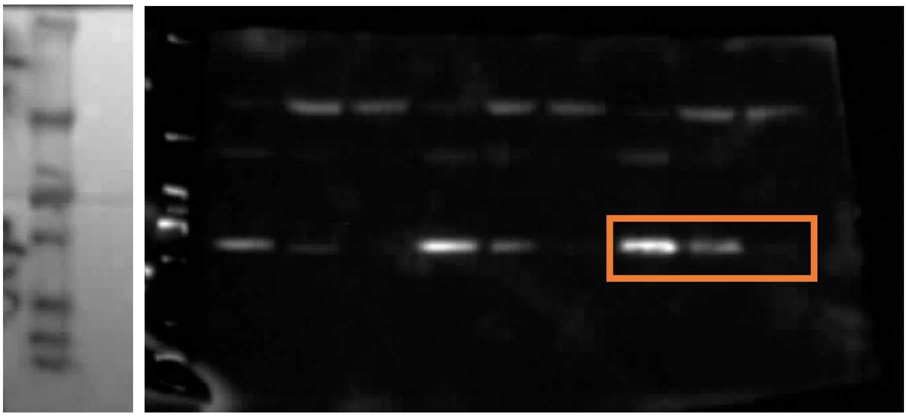

pS6

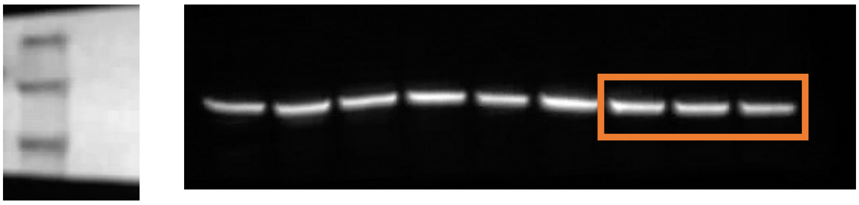

vinculin

Detroit –562 (n=3)

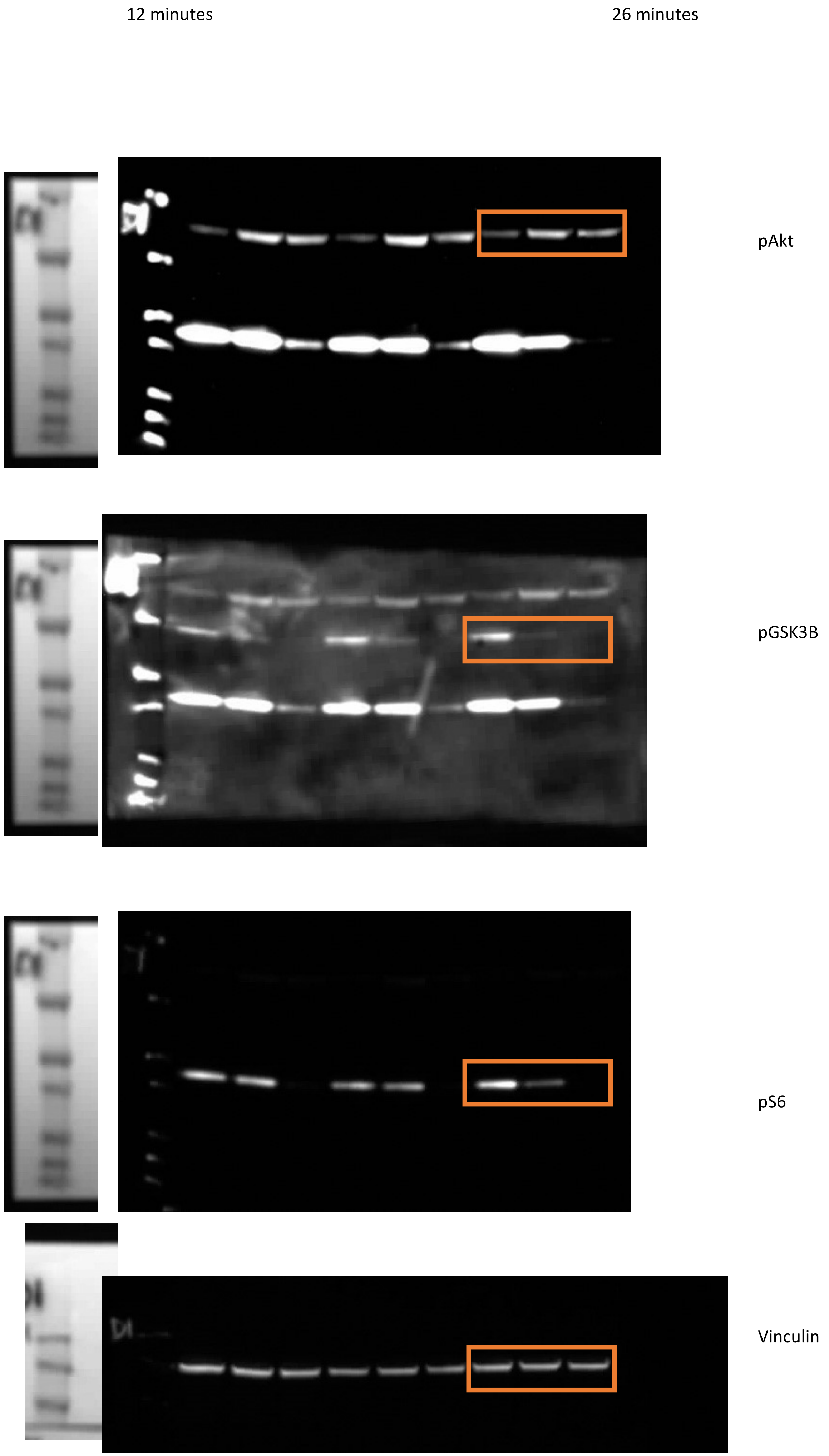

PE/CA PJ15 (n=3)

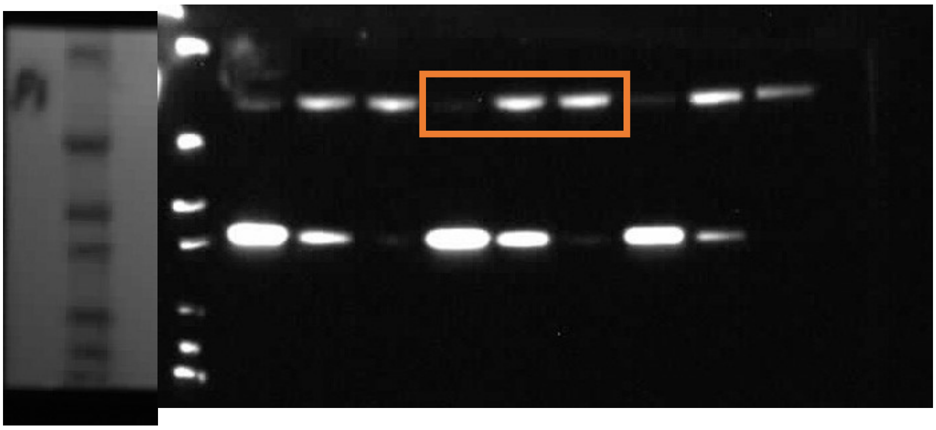

pAkt

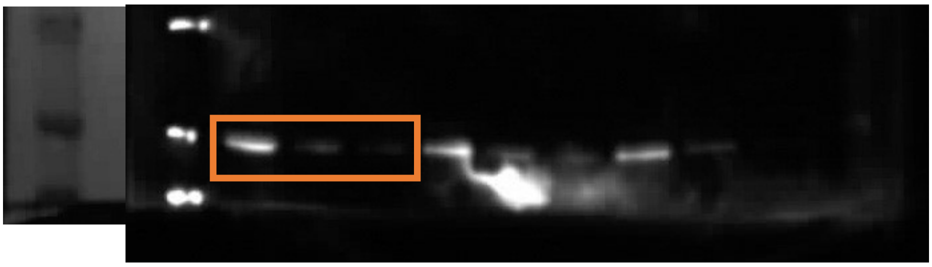

pGSK3B

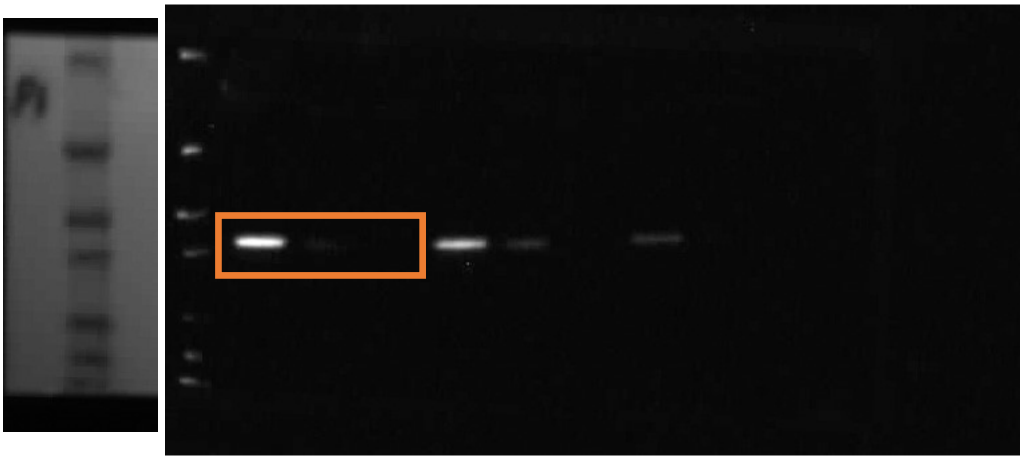

pS6

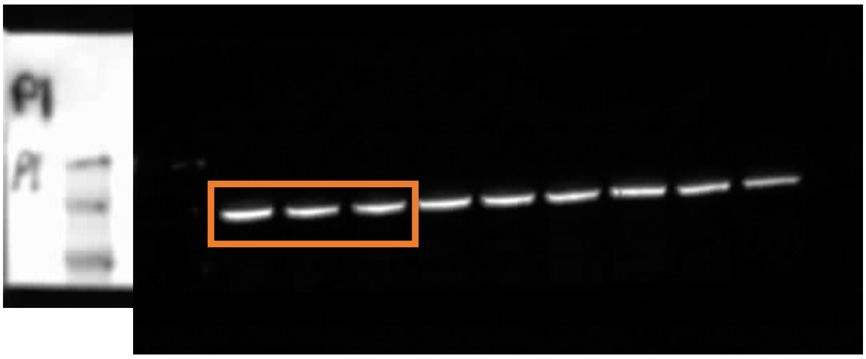

vinculin

RPMI 2650 (n=3)

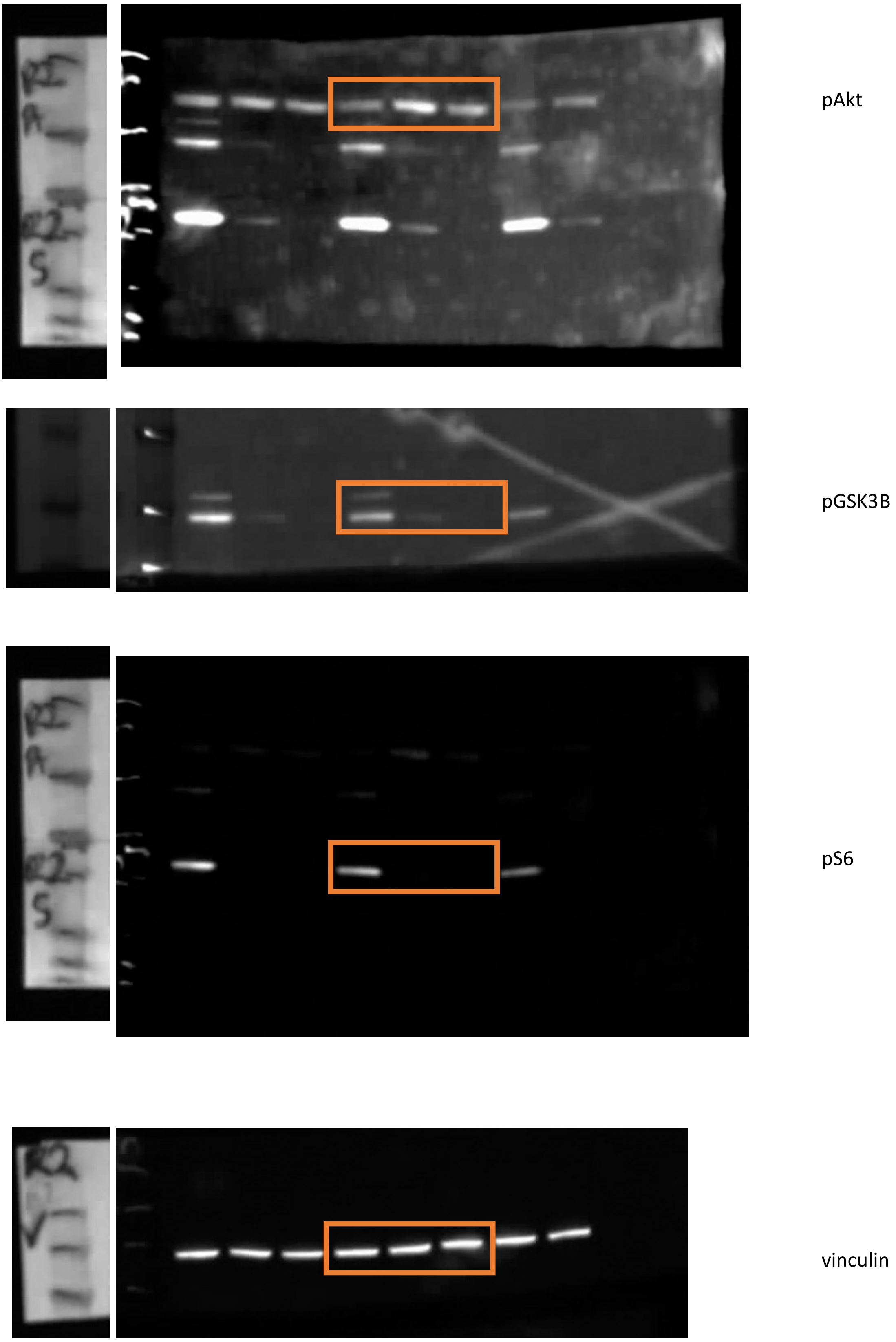

Supplement: Supplementary file 3 — Source Data for Appendix [file EMMM-9-1646-s005.zip › SourceDataAppendixFigS1.pdf]
